# Pooling-analysis for diagnostic and prognostic value of MiRNA-100 in various cancers

**DOI:** 10.18632/oncotarget.18697

**Published:** 2017-06-27

**Authors:** Zhe Dou, Shuai Lin, Cong Dai, Ye Lu, Tian Tian, Meng Wang, Xinghan Liu, Yi Zheng, Peng Xu, Shanli Li, Qianwen Sheng, Yujiao Deng, Zhijun Dai

**Affiliations:** ^1^ Department of Oncology, Second Affiliated Hospital of Xi’an Jiaotong University, Xi’an 710004, China; ^2^ Department of Student Affairs, Second Affiliated Hospital of Xi’an Jiaotong University, Xi’an 710004, China

**Keywords:** miRNA-100, diagnosis, prognosis, meta-analysis

## Abstract

Many studies manifested miRNA-100 was deregulated in various cancers, which indicated that miRNA-100 might be a potential biomarker of cancer diagnosis and prognosis. However, the role of miRNA-100 was still uncertain. We searched for qualified studies using PubMed, EMBASE, Web of Science, Cochrane library and CNKI databases. The diagnostic effect was evaluated by the pooled sensitivity, specificity, and other indexes. Pooled hazard ratios (HRs) with 95% confidence intervals (CIs) for overall survival (OS) were calculated to assess the prognostic value. This meta-analysis included 7 and 19 studies about diagnosis and prognosis, respectively. The results of pooled sensitivity, specificity, positive likelihood ratio (PLR), negative likelihood ratio (NLR) and diagnostic odds ratio (DOR) were 0.75 (95%CI: 0.71-0.78), 0.74 (95%CI: 0.69-0.78), 2.61 (95%CI: 1.81-3.76), 0.33 (95%CI: 0.24-0.45), 8.46 (95%CI: 4.85-14.77), respectively. And, the area under SROC curve (AUC) was 0.8141. We also found that lower expression of miRNA-100 in cancer tissues could significantly predict poorer prognosis in overall cancer (HR = 0.59, 95%CI: 0.39-0.90), especially in genital system tumors (HR = 0.42, 95%CI: 0.27-0.66, P = 0.431), bladder cancer (HR = 0.21, 95%CI: 0.06-0.73, P = 0.143) and esophageal squamous cell carcinoma (HR = 0.26, 95%CI: 0.13-0.52, P = 0.164). Our studies concluded that miRNA-100 has a certain value in diagnosis and it may indicate a poor prognosis of cancers.

## INTRODUCTION

Cancer is always a fearsome disease because of its high mortality. It was estimated by GLOBOCAN that there were about 14.1 million new cancer cases and 8.2 million deaths occurring in 2012 and about 57% of cases and 65% of cancer deaths in developed countries worldwide [1]. Therefore, cancer has become a compelling health problem, and early diagnosis is particularly important in the treatment of cancer, but it is difficult because of the limitations in present diagnostic methods. Imaging examination and biopsy have the disadvantages of their invasive and harmful procedure, and many current biomarkers lack high accuracy in clinical diagnosis. In addition, it’s difficult to predict the clinical outcomes of cancer, which significantly varied in different people. At present, the research of biomarkers has made rapid development [2]. It’s highly needed to seek for new biomarkers that can exert on detection or diagnosis in early-age or estimate the prognosis of patients.

With a length of 19-25 nucleotides, microRNAs (miRNAs) are small non-coding RNAs which could regulate gene expression by blinding 3’ untranslated region (3’UTR) of their target mRNA and inhibiting gene translation. These miRNAs are considered as gene regulators at post transcriptional gene level [3]. As a member of miRNA-99a family, miRNA-100 is located on chromosome11 at 11q24.1 (Gene ID: 406892) and has been demonstrated to play a potential role in cell proliferation, tumorigenesis, angiogenesis and differentiation [4, 5]. Dysregulated expression of miRNA-100 is correlated with cancer diagnosis and prognosis [6]. Many studies suggested miRNA-100 as an oncogene or a tumor suppress gene. However, their conclusions remain controversial. Recent studies demonstrated obviously down-regulated expression of miRNA-100 in many tumor tissues, such as bladder cancer [7, 8], lung cancer [9, 10], esophageal squamous cell carcinoma [11, 12], epithelial ovarian cancer [13, 14], and other cancers [15-19, 20], indicating that it may have a relationship with poorer prognosis in cancer patients. But, evidence from some other studies showed opposite results in several types of cancer [21-25]. In addition, the diagnostic accuracy and the prognostic significance of miRNA-100 remain unclear. With due consideration of the limitations of a single study, we performed this systematic review and meta-analysis to evaluate the diagnostic and prognostic value of miRNA-100 in various cancers.

## RESULTS

### Literature search

**Figure 1:**
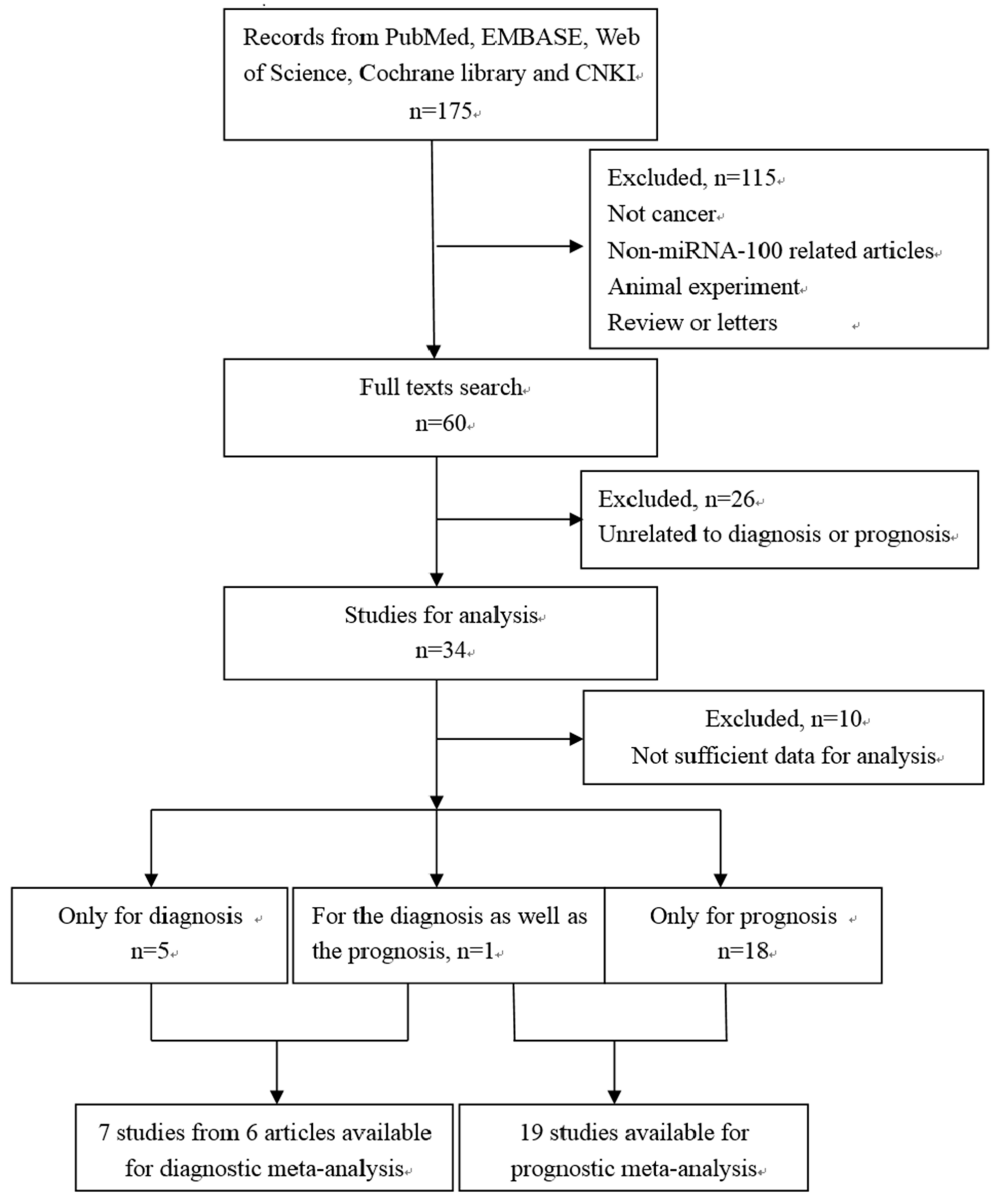
The flow diagram of the study selection process

A total of 175 studies from a primary literature were searched in PubMed, EMBASE, Web of Science, Cochrane library and CNKI. After reviewing titles and abstracts manually, some studies were excluded due to their irrelevance to the analysis, or because they were review articles, duplicate studies, letters, animal experiments or laboratory studies. Then, we reviewed full texts and omitted 26 studies that were unrelated to diagnosis or prognosis and 10 studies without sufficient data to obtain the crucial data for analysis. Finally, 24 available articles were included. Among those articles, one article researched the diagnostic as well as the prognostic value of miRNA-100, meanwhile, it was divided into two studies because of its different investigations in plasma and tissue for diagnosis [16]. One study for prognosis was omitted because of its investigation in serum alone [26]. Finally, we enrolled 19 eligible prognostic studies and 7 eligible diagnostic studies from 6 articles in this meta-analysis (Figure 1).

### Diagnostic meta-analysis

### Study characteristics

**Figure 2:**
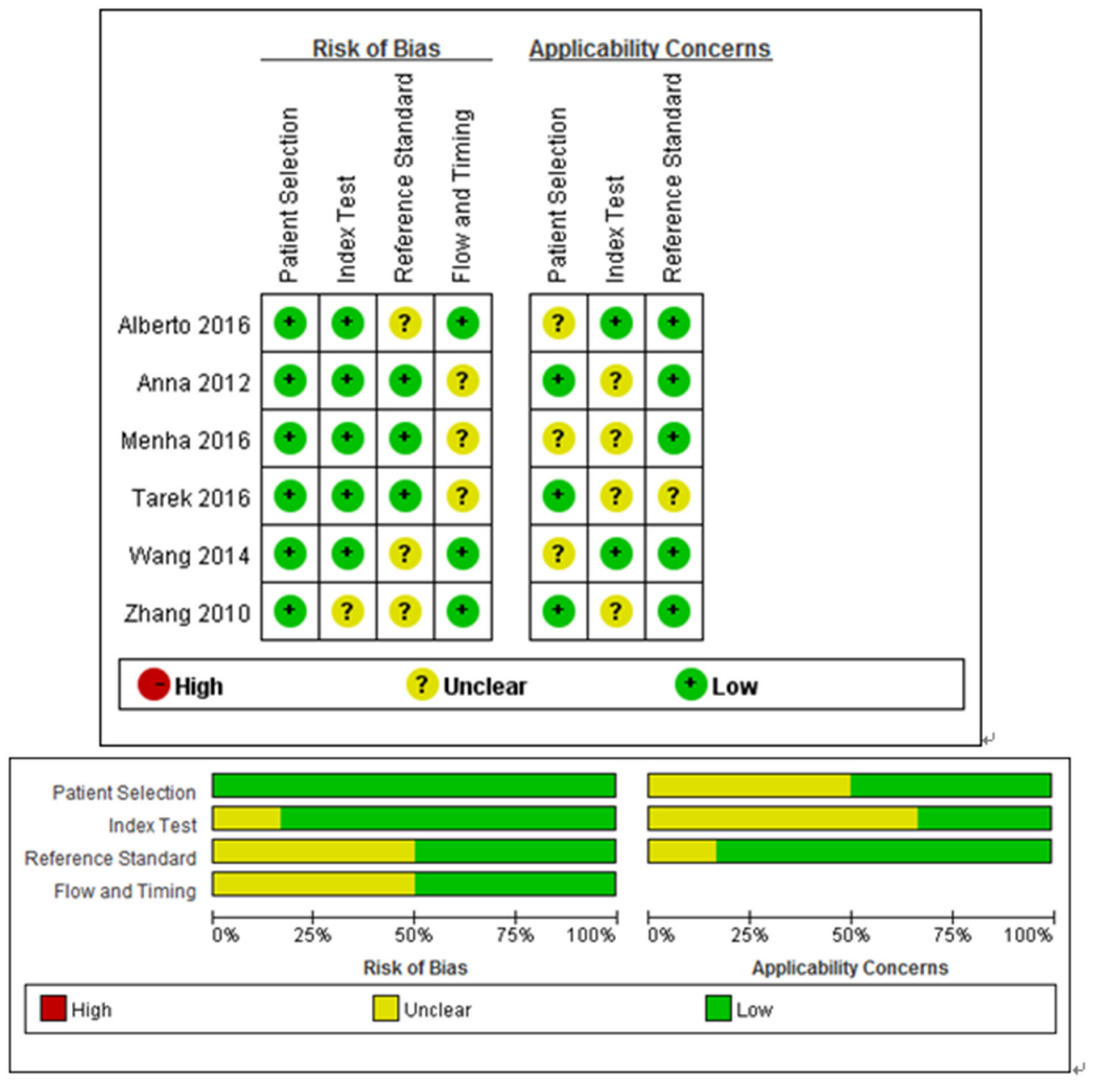
Details of quality assessment by the QUADAS-2 tool “-” in red and “+” in green mean high risk and low risk respectively. “?” in yellow means unclear risk.

7 eligible articles of cancer diagnosis were published from 2010 to 2016, involving a total of 883 participants. These participants were from China, Egypt, Poland, and Mexico. Various types of tumors contain bladder cancer, gastric cancer, endometrioid endometrial carcinoma, esophageal squamous cell carcinoma, acute lymphoblastic leukemia and prostate cancer. Specimens contain serum/plasma, tissue, and urine. And, all studies adopted the approach of quantitative reverse transcription polymerase chain reaction (qRT-PCR) to measure the expression of miRNA-100. The main characteristics of these eligible studies were listed in Table 1 [16, 27-31]. The quality of the studies according to QUADAS-2 tool was good, which was summarized in Figure 2.

**Table 1:** Main characteristics of eligible studies in diagnostic systematic review

Author	Year	Country	Tumor type	Patients	Controls	Specimen	Method	AUC	TP	FP	FN	TN
Tarek et al	2016	Egypt	BC	70	62	serum	qRT-PCR	0.823(0.728-0.917)	63	21	7	41
Wang et al	2014	China	GC	50	47	serum	qRT-PCR	0.71(0.61-0.82)	36	20	15	27
Anna et al	2012	Poland	EEC	34	14	plasma	qRT-PCR	0.740(0.592-0.857)	22	3	12	11
				73	31	tissue	qRT-PCR	0.652(0.548-0.746)	63	16	10	16
Zhang et al	2010	China	ESCC	149	100	serum	qRT-PCR	0.817(0.763-0.870)	95	19	54	81
Menha et al	2016	Egypt	ALL	85	25	serum/plasma	qRT-PCR	0.87(0.779–0.934)	70	0	15	25
Alberto et al	2016	Mexico	prostate cancer	73	70	urine	qRT-PCR	0.738(0.652-0.823)	51	13	22	57

BC = bladder cancer, GC = gastric cancer, EEC = endometrioid endometrial carcinoma, ESCC = esophageal squamous cell carcinoma, ALL = acute lymphoblastic leukemia, qRT-PCR = quantitative reverse transcription polymerase chain reaction, AUC = the area under the SROC curve, TP = true-positive, FP = false-positive, FN = false-negative, TN = true negative.

### Diagnostic accuracy and threshold analysis

**Figure 3:**
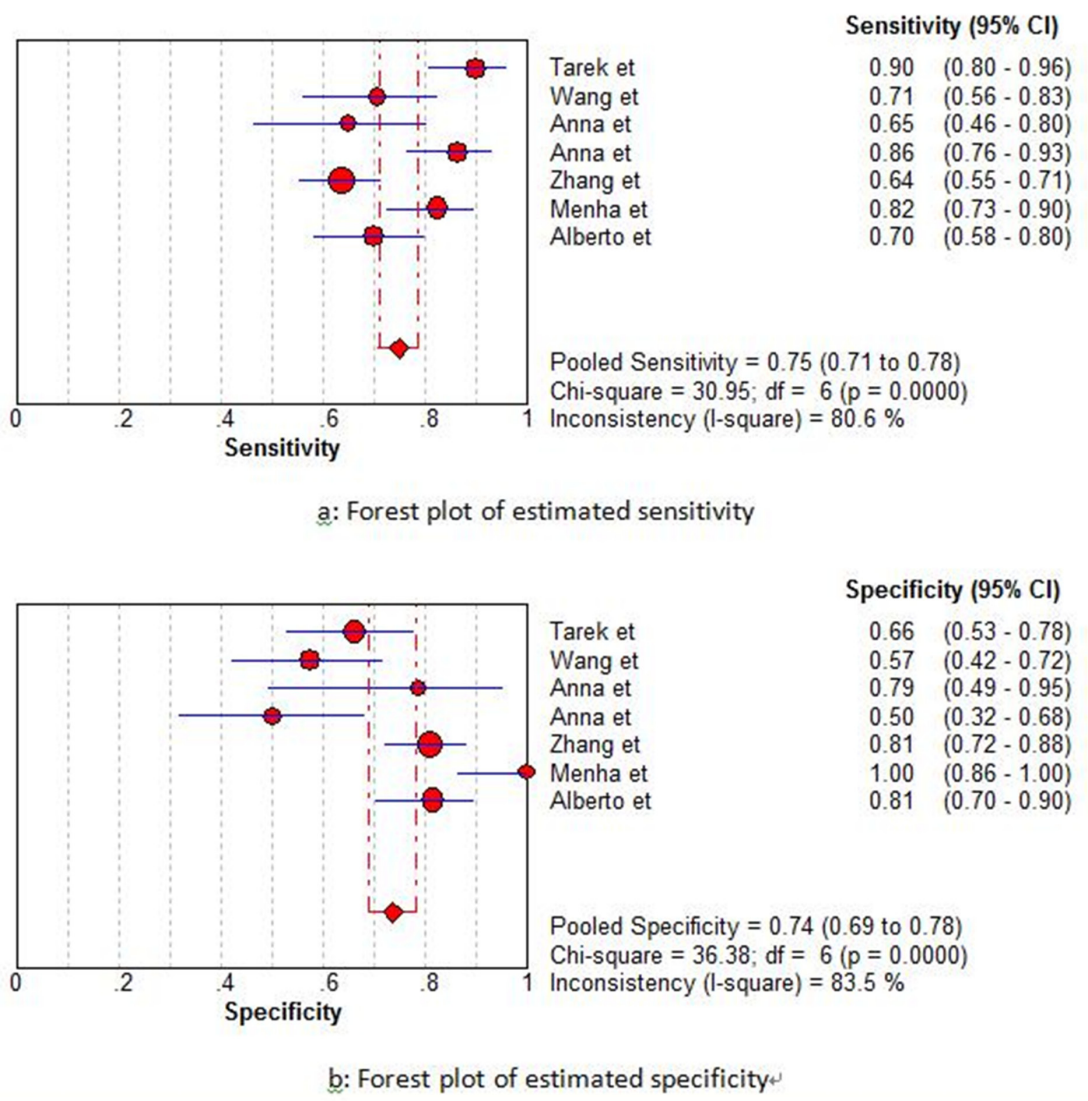
Forest plots of estimated sensitivity (a) and specificity (b) for miRNA-100 in the diagnostic analysis

**Figure 4:**
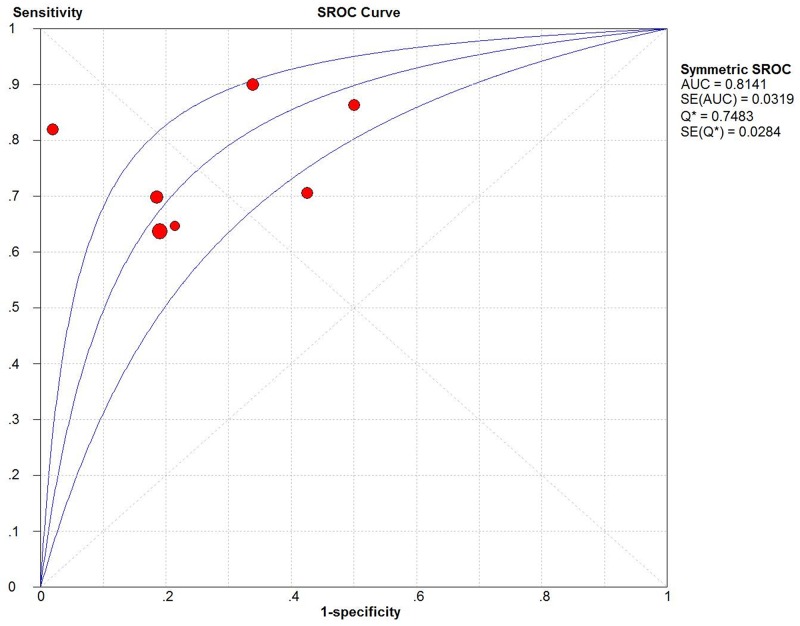
Summary receiver operating characteristic (SROC) Curves of miRNA-100

Firstly, we used the receiver operating characteristic curve (ROC) to identify whether it exist threshold effect. The result showed that there was no heterogeneity from threshold effect. What’s more, the Spearman’s correlation coefficient in this meta-analysis was 0.393 (P= 0.383), which confirmed the result was objective. According to results of the inconsistency index (I^2^), we chose the random-effect model to calculate all indexes. The results of the pooled sensitivity, specificity, positive likelihood ratio (PLR), negative likelihood ratio (NLR), diagnostic odds ratio (DOR) were 0.75 (95%CI: 0.71-0.78), 0.74 (95%CI: 0.69-0.78), 2.61 (95%CI: 1.81-3.76), 0.33 (95%CI: 0.24-0.45), 8.46 (95%CI: 4.85-14.77), respectively (Figure 3). Moreover, as shown in Figure 4, the area under curve (AUC) was 0.8141, suggesting that miRNA-100 had a certain value in diagnosis.

### Prognostic meta-analysis

### Study characteristics

A total of 19 studies with 2009 patients were included in this prognostic meta-analysis. Among those, patients in 16 studies were from China [7-12, 14, 15, 17-21, 23-25] and the other 3 studies were from Germany [22], Poland [16], and Iran [13]. All the studies were published from 2012 to 2016. The tumors types involved to colorectal cancer (n=2), lung cancer (n=3), bladder cancer (n=2), esophageal squamous cell carcinoma (n=2), acute leukemia (n=2), breast cancer (n=1), pancreatic ductaladeno carcinoma (n=1), hepatocellular carcinoma (n=1), renal cell carcinoma (n=1), endometrioid endometrial carcinoma (n=1), small cell carcinoma of the cervix (n=1), and epithelial ovarian cancer (n=2). The numbers of patients ranged from 44 to 204. The expression level of miRNA-100 was measured by qRT-PCR, and HRs and 95%CIs for OS was extracted from each studies. There were 10 studies based on univariate analysis and 9 studies with multivariate analysis. The main characteristics of the eligible studies were listed in Table 2, which also included the scores according to the Newcastle-Ottawa scale (NOS).

**Table 2:** Main characteristics of eligible studies in prognostic systematic review

Author	Year	Country	Tumor type	Sample size	Specimen	Method	Cutoff	Outcomes	Follow-up (months)	Survival analysis	NOS
Susan et al	2016	Iran	EOC	55	tissue	qRT-PCR	-	OS	40(7-90)	U	8
Zhang et al	2015	China	breast cancer	204	tissue	TCGA database	-	OS	10-170	U	8
Zhang et al	2015	China	CRC	172	tissue	qRT-PCR	median	OS	41	U	7
Sameer et al	2015	Germany	PDAC	98	tissue	qRT-PCR	5	OS	0-120	U,M	8
Luo et al	2015	China	NSCLC	48	tissue	qRT-PCR	median	OS	18	U	7
Cao et al	2015	China	BC	92	tissue	qRT-PCR	-	OS	0-50	U,M	7
Zhou et al	2014	China	ESCC	120	tissue	qRT-PCR	median (1.77)	OS	22.62(2.63-76.87)	U,M	8
Chen et al	2014	China	CRC	138	tissue	qRT-PCR	median (1.26)	OS	5-60	U.M	8
Li et al	2013	China	ALL	111	bone marrow	qRT-PCR	-	OS	0-60	U	8
Chen et al	2013	China	HCC	134	tissue	qRT-PCR	-	OS	0-60	U,M	7
Wang et al	2013	China	RCC	96	tissue	qRT-PCR	median (5.5)	OS	81.8(25.2–133.6)	U,M	8
Sun et al	2013	China	ESCC	61	tissue	qRT-PCR	-	OS	0-100	U	7
Wang et al	2012	China	NSCLC	92	tissue	qRT-PCR	median (0.02)	OS	6 (1-33)	U,M	7
Anna et al	2012	Poland	EEC	104	tissue	qRT-PCR	-	OS	10-150	U	7
Wang et al	2012	China	BC	126	tissue	qRT-PCR	-	OS	36	U,M	7
Huang et al	2012	China	SCCC	44	tissue	qRT-PCR	6.515	OS	23.6(2-70)	U,M	7
Peng et al	2012	China	EOC	98	tissue	qRT-PCR	median (0.14)	OS	0-60	U	8
Liu et al	2012	China	NSCLC	110	tissue	qRT-PCR	-	OS	0-65	U	7
Bai et al	2012	China	AML	106	bone marrow	qRT-PCR	median (10.8)	OS	35(10-86)	U	8

M = multivariate, U = univariate, qRT-PCR = quantitative reverse transcription polymerase chain reaction, CRC = colorectal cancer, PDAC = pancreatic ductal adenocarcinoma, NSCLC = non small cell lung cancer, BC = bladder cancer, EEC = endometrioid endometrial carcinoma, ESCC = esophageal squamous cell carcinoma, AML = acute myelocytic leukemia, ALL = acute lymphoblastic leukemia, HCC = hepatocellular carcinoma, RCC = renal cell carcinoma, GC = gastric cancer, SCCC = small cell carcinoma of the cervix, EOC = epithelial ovarian cancer, OS = overall survival, NOS = Newcastle-Ottawa scale.

### Meta-analysis and subgroup analysis

**Figure 5:**
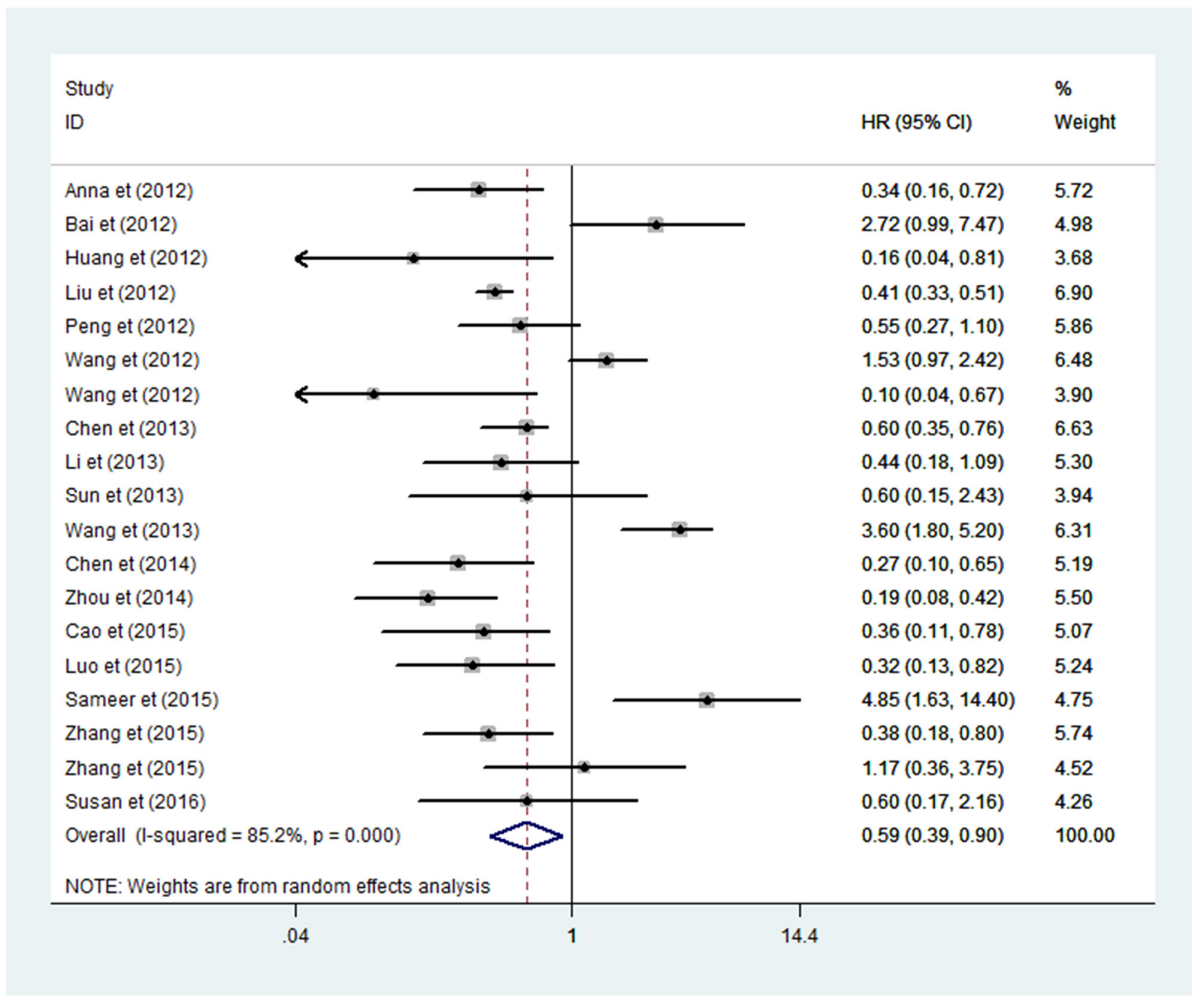
Forrest plots of studies evaluating HRs of high miRNA-100 expression as compared to low expression for cancer CI = confidence interval, HR = hazard ratio.

Obvious heterogeneity was found among these 19 studies for the correlation between the expression of miRNA-100 and overall survival (OS) (I^2^ = 85.2%), so we used the random-effect model to combine hazard ratio (HR) value and 95%CI. With a pooled HR for OS of 0.59 (95%CI: 0.39-0.90), our findings demonstrated that decreased expression of miRNA-100 in tissue predicted a poor clinical outcome (Figure 5). Likewise, the subgroup analysis was integrated into the investigation of heterogeneous sources and the relationship between HRs value and other variables, including sample size, types of cancers, methods, and countries (Table 3). Apparently, we found a significant relationship with lower HR in genital system tumors (HR=0.42, 95%CI: 0.27-0.66 P=0.431), bladder cancer (HR = 0.21, 95%CI: 0.06-0.73, P = 0.143) and esophageal squamous cell carcinoma (HR = 0.26, 95%CI: 0.13-0.52, P = 0.164). Moreover, the results showed there was obvious statistical significance among Chinese subjects (HR = 0.55, 95%CI: 0.35-0.86) and for studies with larger sample sizes (>100 subjects) (HR = 0.44, 95%CI: 0.30-0.64). Considering the difference between analysis methods, we conducted subgroup analysis by analysis methods, showing that the results were meaningful among the studies used univariate analyses (HR = 0.56, 95%CI: 0.38-0.82, shown in Table 3).

**Table 3:** Main results of the pooled analysis

Survival	Variables	No. of studies	Rondom-effects model or fixed-effects model			Heterogeneity	
			No. of patients	Pooled HR	95%CI	I^2^	P
OS	All	19	2009	0.59	0.39-0.90	85.20%	0.000
	Type						
	genital system tumors	4	301	0.42	0.27-0.66	0.00%	0.431
	digestive system	6	723	0.65	0.29-1.47	80.40%	0.000
	respiratory system	3	250	0.60	0.23-1.62	92.60%	0.000
	urinary system	3	314	0.54	0.04-4.74	93.90%	0.000
	others	3	421	0.74	0.23-2.37	80.9%	0.005
	Sample						
	>100	10	1325	0.44	0.30-0.64	67.70%	0.001
	<100	9	684	0.83	0.41-1.70	84.40%	0.000
	Country						
	China	16	1752	0.55	0.35-0.86	85.80%	0.000
	Other countries	3	257	0.98	0.19-5.10	87.20%	0.000
	Method						
	Univariate	10	1069	0.56	0.38-0.82	52.40%	0.032
	Multivariate	9	940	0.57	0.28-1.17	89.80%	0.000

### Meta aggression and sensitivity analysis

We conducted the meta-regression based on publication year, country, sample size, analysis method, tumor type and follow-up period, with an intention of exploring the potential source of heterogeneity in our analysis. However, there was no obvious evident revealed from the results that either of the above covariates in this meta-regression contributed to heterogeneity (shown in Table 4).

**Table 4:** Meta-regression analyses of potential source of heterogeneity

Heterogeneity factors	Coefficient	SE	Z	p	95% CI	
					LL	UL
Publication year	0.029	0.181	0.16	0.875	-0.354	0.412
Country	0.565	0.661	0.85	0.404	-0.829	1.960
Number of patients	-0.001	0.006	-0.16	0.876	-0.144	0.012
Analysis method	0.400	0.472	0.85	0.408	-0.595	1.396
Tumor types	-0.106	0.140	-0.76	0.459	-0.402	0.189
Follow-up	0.006	0.006	1.07	0.299	-0,006	0.018

SE = standard error, CI = confidence interval, LL = lower limit, UL = upper limit.

**Figure 6:**
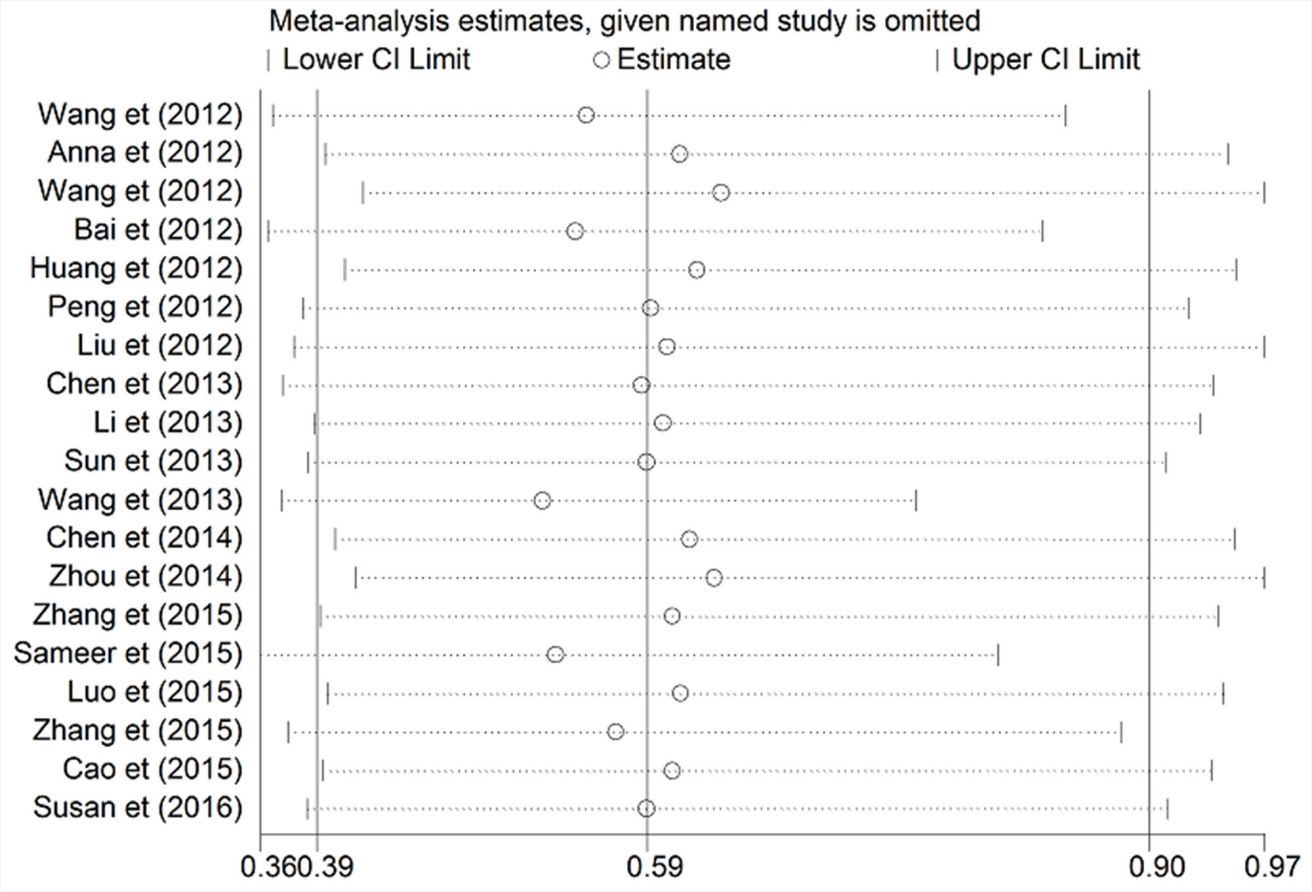
Sensitivity analysis on the pooled hazard ratio for miRNA-100 and overall survival of patients

Meanwhile, we performed sensitivity analysis on the pooled HR for OS about the expression of miRNA-100 in patients. The selected studies were sequentially removed to investigate whether any single study could have an influence on the pooled HRs. As displayed in Figure 6, the results were stable and not significantly affected by each individual study.

### Publication bias

**Figure 7:**
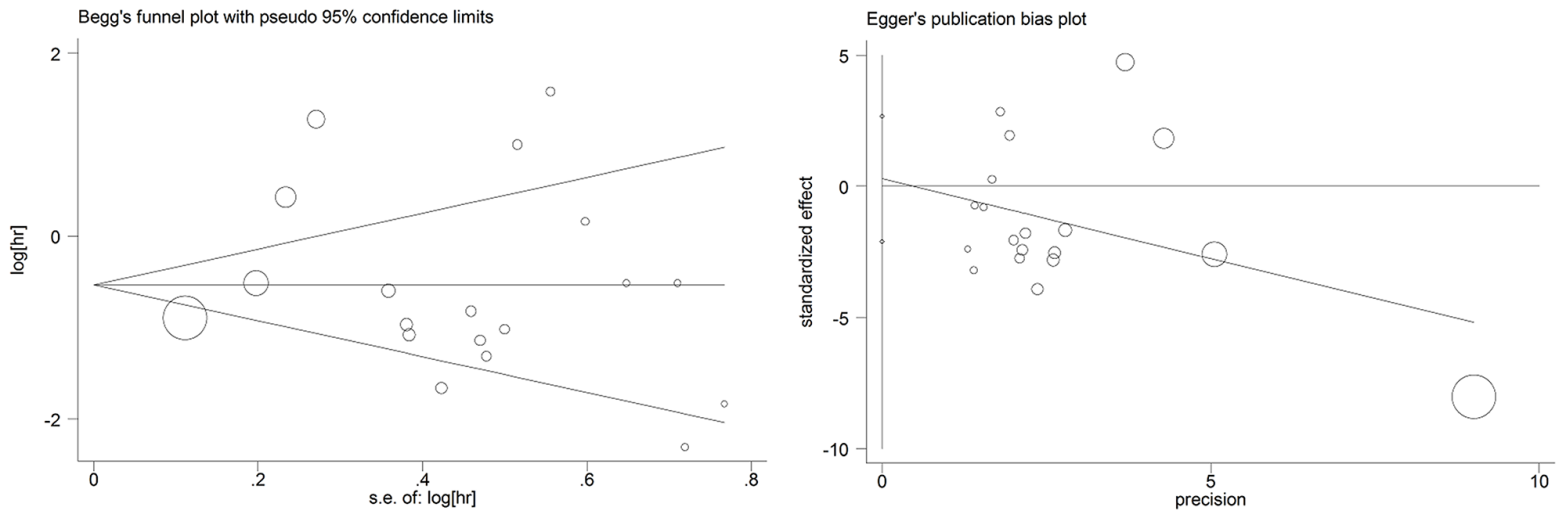
Begg’s and egger’s funnel plots for all of the included studies reported with overall survival

Begg’s and Egger’s tests were used to evaluate the publication bias of the included studies (Figure 7). Begg’s funnel plot did not reveal any evidence of significant asymmetry. With the P value of Egger’s test of being 0.800, it indicated no significant existence of publication bias.

## DISCUSSION

Many investigators reported miRNA-100 in various cancers as a novel molecular target. And, mammalian target of rapamycin (mTOR) gene and insulin-like growth factor 1 receptor (IGF1R) are direct target of miRNA-100 in bladder cancer, acute myelocytic leukemia, endometrioid endometrial carcinoma and so on [12, 16, 20, 32]. MiRNA-100 could suppress the related proteins of the IGF/mTOR signaling cascade in different cancers. Overexpression of miR-100 inhibited the expressions of IGF1R and mTOR by targeting its 3′-UTR at posttranscriptional gene level, so that interfer cell proliferation and survival signaling in some types of tumors. And, miRNA-100 can also exert as a tumor suppressor in many cancers by targeting polo-like kinase 1 (PLK1) [9, 14, 19]. MiRNA-100 was found to significantly inhibit the expression of PLK1 and other proteins, which has a vital effect on cell growth, apoptosis, development and drug resistance. In addition, several studies reported that miR-100 regulated apoptosis in gastric tumor cells and breast cancer cells [33, 34], and they declared miR-100 antagonism triggers apoptosis by inhibiting ubiquitination-mediated p53 degradation [35]. Besides, Cyr61 and RBSP3 was discovered a potential target of miRNA-100 for regulation. Hence, these miRNA-100 related cellular and molecular pathways may provide some ideas for new therapeutic targets in many types of cancers.

The present meta-analysis for diagnosis showed us a pooled sensitivity of 0.75, a pooled specificity of 0.74, DOR of 8.46, AUC of 0.1841, and a PLR of 2.61, which illustrated that there was an approximately 3-fold higher possibility of being miRNA-100 positive for patients with cancer in comparison to those without. And, a NLR of 0.33 mean the probability of miRNA-100 negative patients having cancers was 33%, which suggested that the diagnosis of miRNA-100 existed a certain degree of accuracy but not high enough. But it still had a great advantage compared to other traditional serum-based biomarkers, such as the sensitivities for lung cancer of CEA, Cyfra21-1, SCC and NSE were 46.2%, 40.0%, 43.1%, and 46.2%, respectively. The clinical significance of single biomarker was not ideal. So it may achieve a better diagnostic accuracy through uniting other biomarkers or clinical examinations.

For prognostic value, some studies indicated the low miR-100 expression in bladder cancer predicts unfavorable prognosis and it might regulate tumor metastasis or other related processes about tumorigenesis by inhabiting mTOR [36-41]. A study in EEC observed that decreased miRNA-100 in EEC tissues and up-regulated miRNA-100 in plasma by targeting mTOR making it as a promising biomarker for diagnosis and prognosis [16]. Some studies on EOC, SCCC and NSCLC found miR-100 was significantly decreased in cancer tissues in comparison to healthy people, which appeared that low miR-100 was a poor prognostic biomarker by targeting PLK1 in patients [9, 11, 13, 14, 19, 42] and some researches in HCC, CRC and ESCC hold the similar view [11, 12, 17, 43-45]. But, there are also some studies had different opinions in some types of cancer. Some studies showed miRNA-100 was up-regulated in cancer tissues of AML, RCC, PDAC and NSCLC, causing the result was quite opposite [21-23, 46]. Then, we conducted this meta-analysis for prognosis, and we found lower miRNA-100 expression may predict a poorer outcome in various cancers, and the predictive efficacy was more significant in genital system tumors, BC and ESCC. HRs were significant for studies in Chinese subjects, larger sample sizes (>100 subjects) and by univariate analyses. So we think miRNA-100 may be a potential biomarker for prognostic. However, as the results of sensitivity analysis and meta-regression, we couldn’t find the resource of heterogeneity, so we summarized the data using the random-effect model. Considering the source of heterogeneity, we speculated that the heterogeneity may be caused by the cut-off value of miRNA-100 expression, which was not been reported explicit values in many articles, and multiple factors may influence the heterogeneity together, such as the difference of selection criteria for patients in various tumor types and studies, the diversified clinicopathological characteristics of patients in different studies, the specific method of randomization and blind, the random errors in studies and so on.

Recently, a meta-analysis was reported about prognostic value of miRNA-100 in cancers. They suggested that patients with lower expression of miRNA-100 in cancer tissue had poorer survival in a variety of carcinomas, which was similar to our result in prognostic meta-analysis [47]. There have many differences between us, such as published time, sample size, statistical software, data processing and so on. Firstly, six studies published before October 2013 were included in that study, while we extracted data from 19 available studies published before October 2016 for prognostic and 7 studies for diagnostic meta-analysis. Secondly, in the measures of data processing, they only calculated the pooled HR for OS and investigated publication bias in their analysis, without exploring the source of heterogeneity in that study. However, we carried out the subgroup analysis by some variables and discovered that there were significant results especially in genital system tumors, bladder cancer and esophageal squamous cell carcinoma. Meanwhile, with an aim of probing deeply into the source of heterogeneity, we executed the meta-regression, subgroup analysis and sensitivity analysis. What’s more, the diagnostic meta-analysis was also referred to the investigation of the diagnostic and prognostic significance ofmiRNA-100 in various cancers. So, we have more advantages in comparison with previous study. Our study is the first meta-analysis to research the diagnostic and prognostic value of miRNA-100 in various cancers. In addition, our study may be more comprehensive and abundant due to our efforts in subgroup analysis, meta-regression and so on. Besides, many new studies have been incorporated into our article, which contributed to a more reliable conclusion.

Nevertheless, this study still exists some limitations. Firstly, research and sample size in single tumor type was relatively small, which probably influenced the research in single cancer. Secondly, our studies have a very high ratio of data in Chinese patients, which may limits its application to global range. Thirdly, some HRs could not be extracted from primary studies and needed to calculate from the Kaplan-Meier survival curves using indirectly method, which may cause a certain calculation error. Finally, it exists an obvious heterogeneity in our meta-analysis, according to the sensitivity analysis and meta-regression, and we could just supposed the resource of heterogeneity. Therefore, further studies in the future are expected to draw a more definitive conclusion.

In summary, we concluded miRNA-100 had a certain value in diagnosis despites its diagnostic accuracy was not high enough, and it had a significant value as a prognostic biomarker. Therefore, investigating the expression of miRNA-100 in various cancers may provide a new thinking into cancer prevention and therapeutic strategy, and the different expression level of miRNA-100 in cancers may indicate different endings of patients.

## METHODS

### Search strategy and selection criteria

This meta-analysis was conducted following the guidelines of the Meta-analysis of Observational Studies in Epidemiology and Preferred Reporting Items for Systematic Reviews and Meta-Analyses groups [48]. We carefully searched for the relevant articles in PubMed, Web of Science, EMBASE and CNKI (up to October 31, 2016) assessing the diagnostic accuracy and the prognostic significance of miRNA-100 in various types of cancers. The keywords such as microRNA-100/miRNA-100/miR-100, cancer/carcinomas, prognosis and diagnosis were used. Moreover, the reference articles from all associated articles were also found and scanned manually to retrieve any additional eligible studies.

The eligible studies must fit the following inclusion criteria: (i) the study investigates the diagnostic or prognostic value of miRNA-100 in patients with various carcinomas; (ii) for diagnosis, they must provide enough information that we could obtain the crucial data directly or through calculation, such as true-positive (TP), false-positive (FP), false-negative (FN), and true negative (TN); (iii) for prognosis, they must provide enough information so that we could extract directly or indirectly HRs with 95% CIs for OS; (iv) for prognosis, they measured the expression of miRNA-100 in tumor tissues.

Articles will be excluded by following criteria: (i) duplicate studies; (ii) review articles or letters; (iii) non-original articles; (iv) animal experiments and laboratory studies. Two reviewers independently searched and identified all articles, resolving disagreements by consensus in research group.

### Data extraction

Two investigators independently made judgments and extraction of the relevant data, settling disagreements through consensus adjudication by research group. The extracted data included name of the first author, publication year, number of patients, cancer types, specimen, test method, diagnostic results (AUC, TP, FP, TN, FN) and related data for prognostic (cut-off, follow-up, HR, 95%CI). If not obtaining diagnostic results directly, we calculate the data using their sensitivity and specificity. We collect HRs and their 95% CIs preferentially from multivariate or univariate analyses in the original article, and HR>1 means higher expression of miRNA-100 in tumor tissues that may have a poorer prognosis in cancer patients. If not available, we calculate HRs with corresponding 95% CIs from Kaplan–Meier curves through Engauge 4.0 software. Meanwhile, study quality of studies in diagnostic meta-analysis was rated by Quality Assessment of Diagnostic Accuracy Studies (QUADAS-2) assessment tool. We also use the Newcastle-Ottawa (NOS) scale to evaluate the quality of each included study, which score ranges from “0” to “9” and a score ≥6 indicates high quality.

### Statistical analysis

As for diagnostic meta-analysis, we calculated and combined sensitivity, specificity, PLR, NLR, DOR, and corresponding 95% 95% CIs based on the key data (TP, FP, FN, TN), ROC and Spearman correlation coefficient were applied to verify a threshold effect. We measured the heterogeneity by the I2. I2> 50% indicated that significant heterogeneity exists in studies, then we implemented the random-effect model to calculate the related indexes (DerSimonian-Laird method), otherwise, the fixed-effect model was selected (Mantel-Haenszel method). Simultaneously, the diagnostic accuracy was assessed by the area under the SROC curve (AUC) from summary receiver operative curve (SROC). In addition, in the case of two-sided p values across the board, P < 0.05 was considered statistical significant. The diagnostic meta-analysis were performed with Meta- Disc software, version 1.4 (Unit of Clinical Biostatistics, Ramony 94 Cajal Hospital, Madrid, Spain) [49].

For prognostic meta-analysis, HRs and 95%CIs of OS were weighted and pooled to estimate the contact between expression of miRNA-100 and prognostic significance in various cancer patients. Higgins I-squared statistic was used to measure statistical heterogeneity, we adopted random-effect model for severe heterogeneity with I2>50%, while fixed-effect model for the absence of heterogeneity with I2<50% [50]. The confounder contribution to heterogeneity was explored through the approach of subgroup analysis, meta-regression and sensitivity analysis. Besides, we adopted Begg’s and Egger’s test to study whether it exists publication bias. All the results were considered statistical significant at two-sided P-value of 0.05. The prognostic meta-analysis was conducted with STATA statistical software, version 14.0 (Stata Corporation, College Station, TX, USA).
